# Pro-ecological and conservation activities are not always beneficial to nature: a case study of two lowland streams in Central Europe

**DOI:** 10.1038/s41598-023-42555-7

**Published:** 2023-09-20

**Authors:** Janusz Golski, Wojciech Andrzejewski, Maria Urbańska, Sławomir Runowski, Krzysztof Dajewski, Lilianna Hoffmann

**Affiliations:** Department of Zoology, Poznań University of Life Sciences, Wojska Polskiego 28, 60-637 Poznan, Poland

**Keywords:** Ecology, Ecology, Environmental sciences, Hydrology, Limnology

## Abstract

Since 1990 and in particular, after the implementation of the Water Frame Directive, many positive effects of pro-ecological projects are evident; unfortunately, examples of adverse effects have also been observed. This study aims to indicate how some ill-considered actions, called “pro-ecological”, may lead to habitat degradation and the disappearance of valuable hydrobiont species. Two watercourses, representing the lowland gravel stream and sandy stream type, were selected for the study. Literature indicated that in the past, these watercourses were characterized by an excellent ecological status and the presence of valuable rheophilic fauna and flora. Environmental parameters were recorded, macroinvertebrates and ichthyofauna were sampled and analyzed, and finally, indexes were calculated. The results were compared with literature data. In the course of studies conducted in 2011–2015, drastic habitat deterioration and extensive changes in the species structure of ichthyofauna and aquatic invertebrates were observed. Changes in the Smolnica stream have been caused by the three retention basins constructed in 2000, along the lower and middle course; while in Kiszewko, however, the factor for habitat deterioration was connected with the excessive expansion of the Eurasian beaver (*Castor fiber*), which created a beaver pond 20 m in width, with impoundment elevations of up to 2 m.

## Introduction

The negative environmental impact of human activity was particularly evident in the second half of the twentieth century. As a consequence of the rapidly increasing population and the related development of conurbations, industry, and agriculture, the resulting dramatically magnified anthropopressure has affected all elements of the biosphere^1–6^.

Numerous studies have shown that by the beginning of the twenty-first century, over 50% of the world’s ecosystems had been degraded by human activity^7^. According to the European Red List of Freshwater Fishes^2^, among all animal groups, it is fish that are most susceptible to negative impacts, with over 40% of species considered to be threatened. Awareness of the threat to the natural environment and the need to counter this process appeared as early as the late 1960s after the report “Problems of the human environment” by U. Thant; however, for many years, the actual effectiveness of the actions undertaken was limited^8–12^.

Since the transformation of the political and economic system after the fall of communism in the early 1990s in Central Europe, we have been observing a gradual increase in ecological awareness along with various ecologically-oriented actions aimed at improving environmental quality^13–16^. These actions also concern aquatic ecosystems and focus, for example on the protection of valuable, endangered habitats and species, water retention, and improving water quality, with their number and scope increasing greatly since the accession of the former communist countries to the European Union in 2004 and the implementation of the Water Framework Directive.

The positive effects of ecologically-oriented actions are evident; unfortunately, we may also find examples of negative effects resulting from insufficient knowledge and the failure to comprise all components of the natural environment^9,10,17,18^. Among decision-makers, we may frequently observe confusion concerning many concepts related to environmental protection, including nature conservation. This may lead to counter-effective actions, such as, e.g., the protection and promotion of one element of the environment, resulting in the deterioration of another. Activities labeled as eco-friendly may not always effectively protect all ecosystem elements^19,20^.

Counter-effective actions harmful to the environment include, for example, building small hydroelectric power plants on rivers that are particularly valuable in terms of nature, introducing alien species (especially fish, crayfish, and birds), and excessive protection of species that have a strong impact on other populations^18,20–24^.

Our observations indicate that this problem often affects lotic ecosystems, as evidenced by the two streams described in this study. Towards the end of the twentieth century, within the program's framework to increase water retention in forest areas, four retention basins were constructed along the lower and middle course of a small Smolnica stream. In Strumień Kiszewski, the population of the Eurasian beaver (*Castor fiber* L.), reintroduced in the later 1980s, increased rapidly. Both these phenomena were connected with the adverse habitat changes observed.

The idea behind the creation of artificial dams and the reintroduction of beavers which also dam the streams was to increase the retention in the forest and restore the endangered species, but the decision-makers did not take into account the fact that each dam, regardless they are artificial or built by beavers, changes the environmental conditions in the adjacent sections.

Disturbed river continuum is considered to be a major factor negatively affecting species diversity and the potential for survival of valuable species^5,8,11,25–29^. Hydraulic structures indirectly and directly affect the ecological status and use the value of waters upstream and downstream of the damming. The most significant negative effects include changes in the character of these waters from flowing to stagnant; sanding, silting, or leaching of fine-grained fractions; fluctuations in water levels, reduction of microhabitat diversity; an increase in temperature and a decrease in oxygen content in the summer period; and deterioration of photosynthetic conditions^12,19,26,30–32^. In the cases described, lowland streams were transformed into a sequence of lenitic reservoirs and short semi-natural lotic sections or a series of widely spread ponds with a homogeneous flow and muddy bottom.

Among the positive effects of beaver activity, the most frequently mentioned aspects include increasing habitat diversity thanks to the division of the watercourse into lentic and lotic stretches, an increased density of invertebrates^33–36^, as well as reduced sediment content, including that of some biogenic^37–39^.

Negative opinions on beaver ponds are connected mainly with sanding and silting of their bottoms, deterioration of physicochemical parameters, and water stagnation^33,38,40–42^. In lowland rivers and streams, the formed ponds additionally cause an increase in temperature and a decrease in oxygen content in the summer period^40,41,43–45^. In South America (Patagonia), the beaver is considered an invasive species, destroying the landscape by eliminating the forested riparian zone and digging out considerable amounts of sediments and peat soils^46^.

This study aims to indicate how these ill-considered actions, focused only on one aspect of the environment, i.e., misjudged localization of technical small retention facilities and excessive species protection of the Eurasian beaver, may lead to habitat changes or even degradation and depletion of valuable plant and animal species. To achieve the aim of the research, the authors put forward the following research hypotheses: (1) the presence of retention reservoirs in small lowland streams causes far-reaching, negative changes in abiotic and biotic factors; (2) the uncontrolled growth of the European beaver population in small lowland streams causes far-reaching, negative changes in abiotic and biotic factors.

## Material and methods

**Figure 1:**
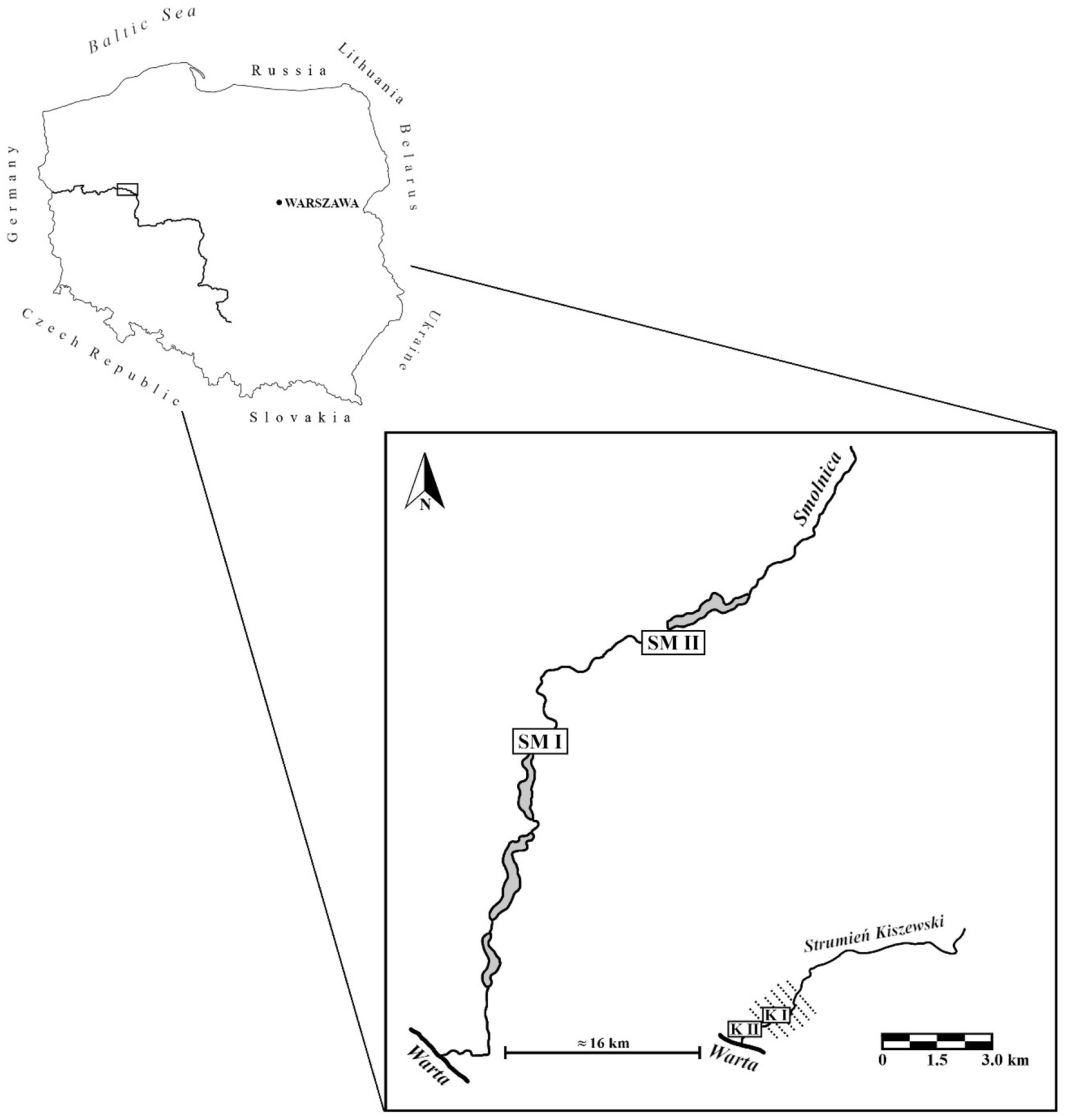
Location of the research site. Boxes with abbreviations indicate the location of the sections where the research was conducted, while the dotted lines indicate the location of the beaver dams.

Strumień Kiszewski and Smolnica, two lowland streams located in the Natura 2000 Area: Puszcza Notecka PLB300015 (the Wielkopolskie province, Poland), flowing to the Warta River near Kiszewo and Wronki, were studied (Fig. 1). The Natura 2000 network consists of protected, naturally valuable areas located in the territory of the European Union^47^.

The foundation for the decision to undertake this study was provided by a publication by Iwaszkiewicz^48^, which stated that these small lowland streams, i.e., Strumień Kiszewski and Smolnica, were characterized by an excellent ecological status and the presence of valuable, sensitive to environmental condition rheophilic fauna and flora. Unpublished data from research catches conducted in the early 1990s (Andrzejewski et al. unpublished data) were also found and used. These data included environmental conditions and fish densities from a single survey.

Research conducted in 2011–2015 consisted of three stages: determination of parameters characterizing environmental conditions, collection of benthos samples, and catches of ichthyofauna. Each stream was divided into two sections differing in their modification rate (Fig. 1).

In Strumień Kiszewski (52.698974 N; 16.639779 E), analyses were conducted in the area of beaver dams (KI) and on a short reach downstream of the dammed section (KII). In Smolnica (52.747748 N;16.437018 E), the studied sections were located approx. 1 km below a retention basin (SI) and immediately below the retention basin (SII). All selected sections overlap entirely with the stretches studied by Iwaszkiewicz^48^, while research points characterized a given section.

Hydromorphological (width, depth, flow) and basic physicochemical (temperature, oxygen content, conductivity, pH) parameters were measured three times a year, while the bottom structure was determined yearly. These studies were repeated every year. Based on the received data, mean stream widths and depths were calculated, along with the ratio of mean width to mean depth and the proportion of gravel areas in the streambed of the analyzed watercourses. Values of temperature and oxygen content are given for the summer period, when environmental conditions are usually the worst, while conductivity and pH are presented for the entire research period (Table 2).

During environmental research, the species composition of aquatic vegetation was also determined, and the dominant species were given.

Every year, in the spring and autumn, samples of benthic macroinvertebrates were collected using a Surber sampler (1000 cm^−2^ sampling area and 0.5 mm mesh size). On each occasion, two samples were taken from shallow and deep water from one station of each section. The density of invertebrates (individuum.m^−2^), dominance structure (percentage share in the number D%), the Shannon–Wiener diversity index, and the BMWP score were calculated. The value of the diversity index is influenced by the number of taxa and the evenness of their share in the abundance. It ranges from 0 (low diversity) to 1 (high diversity)^49,50^. The BMWP score is the biotic index, using indicator taxa of aquatic invertebrates, assigning values to groups, usually families, ranging from 0 to 10, depending on their sensitivity to environmental changes. This index ranges from 0 (bad quality) to over 100 (very good quality)^51^. In the source publication^48^, higher taxonomic groups of invertebrates (class, subclass, ordo, family) have been identified, and therefore similar accuracy has been retained in this paper.

Catches of fish using an impulse fishing device IUP 12 (1.5 kW, 3A, 220 V) were performed every year in September, with all the caught fish released on-site following the identification, counting, and weighing. Each section had a single experimental segment of 200 m in length. During earlier studies, samples were also collected by electrofishing comparably. Fish species were ordered in terms of their affiliation to reproduction groups according to the division proposed by Balon^52^. Fish density was expressed in the number of specimens per 100 square meters of the stream bottom. Based on the collected data, the calculated parameters included the dominance structure (D%), the Shannon–Wiener diversity index (H)^49,50^, as well as the EFI + index. The European Fish Index is an advanced biotic multi-index that uses fish indicator species (sensitive to environmental conditions) to assess the ecological status of watercourses^53^. The system compares the existing values (from the survey) with the reference values characteristic for watercourses not subjected to anthropopressure. It takes values from 0 to 1, which allows the stream to be classified into one of five quality classes—from I (0.911–1.00; very good quality) to V (0.000–0.252; bad quality).

Results were compared to a study by Iwaszkiewicz^48^ and unpublished data collected by the authors in 1992. The species composition of assemblages at individual locations analyzed in 1965 and 2013 was compared using the analysis of faunistic similarity according to the Jaccard formula both in qualitative and quantitative terms^54–56^. The species number and the density of macroinvertebrates and fish at individual sites were used for the analyses. Calculations were conducted using agglomerative hierarchical clustering (complete linkage) with the XLSSTAT 2016 program, and the results were presented as dendrograms.

## Results

### Changes in environmental conditions in analyzed watercourses

Strumień Kiszewski, analyzed in the 1960s by Iwaszkiewicz^48^, was characterized by diverse channel morphometry and slight relative channel shallowing (Tables 1 and 2). A considerable part of the bottom area was covered with gravel, with only a small portion of sand and mud. Aquatic vegetation was represented by watercress (*Nasturtium officinale*) and cutleaf water parsnip (*Berula erecta*). In summer, water temperature did not exceed 13 °C, while oxygen content fluctuated around 10 mgl^−1^ (Table 2).

**Table 1:** Evironmental parameters in analyzed watercourses (catchment parameters).

Parameter	Kiszewko	Smolnica
Total length [km]	4.5	19
Linear length [km]	1.6	5.7
Stream development [%]	35.5	30.0
Catchment [km^2^]	32.1	81.6
Headwaters [m a.s.l.]	69.0	66.5
Mouth [m a.s.l.]	42.7	42.7
Slope [‰]	1.71	0.8

**Table 2:** Changes in environmental parameters in analyzed watercourses in the years 1964–2015 (hydromorphometric and physicochemical parameters).

	Depth [cm] Max./mean	Width [m] Range average	W/D ratio	Mean flow [cm s^−1^]	Bottom substrate [%]	Aquatic vegetation	Catchment land use	Temperature [^o^C]^1^ Range average ± SD	Oxygen [mg l^−1^]^1^ Range average ± SD	pH^2^ Range average ± SD
K 1964	42/26	1.1–1.4 1.2	5	30	G75S20 M5	+ NO, BE	Forests	12.8–13.3 13.1** ± **0.21	9.4–9.8 9.6** ± **0.24	7.6–7.7 7.7 ± 0.06
K* 1992	18/12	1.4–2.0 1.6	13	No data	G35S60 M5	++ ?	Forests	13.2*	8.1*	7.1*
KI 2015	84/46	5.0–21.0 15.0	32	2	M100	+++ LM, CD	Forests	13.8–16.3 15.0 ± 1.22	0.5–2.1 1.3 ± 0.84	6.5–6.6 6.6 ± 0.09
KII 2015	36/17	1.0–1.4 1.2	7	36	G15S80 M5	+ CD, EC	Forests	13.7–14.6 14.1 ± 0.41	5.2–6.1 5.6 ± 0.44	7.1–7.4 7.2 ± 0.13
SM 1964	65/39	1.3–2.4 2.0	5	40	G65S25 M10	+ R	Forests	13.1–13.6 13.3 ± 0.22	9.2–9.8 9.5 ± 0.28	7.7–7.9 7.8 ± 0.09
SM* 1992	50/30	2.2–4.3 3.1	12	50	G60S30 M10	+ ?	Forests	12.0*	9.5*	7.2*
SMI 2015	73/29	2.4–5.0 3.9	12	36	G30S65 M5	+ BE, ETC	Forests	13.9–17.0 15.2 ± 1.35	5.4–7.7 6.8 ± 0.98	6.9–7.4 7.2 ± 0.22
SMII 2015	62/24	2.5–4.7 3.8	16	39	G10S85 M5	+ EC	Forests	16.7–17.8 17.2 ± 0.48	4.9–6.6 5.6 ± 0.74	7.6–7.7 7.6 ± 0.05

W/D ratio—the ratio of mean width to mean depth; bottom substrate: G—gravel, S—sand, M—mud; aquatic vegetation: *NO*—*Nasturtium officinale* (Watercress), BE—*Berula erecta* (Cutleaf water parsnip), *LM*—*Lemna minor* (Common duckweed), *CD*—*Ceratophyllum demersum* (Rigid hornwort), R—*Ranunculus* sp. (Water crowfoots), EC—*Elodea canadensis* (Canadian waterweed).

*In 1992, the environmental parameters were measured only once, in the summer.

^1^Values of temperature and oxygen are given for the summer period.

^2^Values of conductivity and pH for the entire research period.

The next round of studies was conducted in the early 1990s, several years after the reintroduction of the Eurasian beaver. Compared to the previous period, watercourse depth decreased, and simultaneously there was a slight increase in channel width, which resulted in an increase in the W/D ratio. The most significant changes affected the bottom substrate, in which gravel content decreased markedly while amounts of sand and mud increased. The values of physicochemical parameters were close to those given by Iwaszkiewicz^48^ (Table 2).

Drastic changes in environmental conditions were observed during the last study, over twenty years after beaver reintroduction (Table 2). Over almost the entire watercourse length in the KI section, numerous beaver dams over 1.5 m in height were located. As a result, the mean watercourse width increased more than 12-fold, while depth increased twofold in relation to output data. An almost imperceptible water flow was observed. The entire bottom area was covered by a layer of organic matter which was max. 0.5 m deep. Aquatic vegetation was found in considerable densities and was represented by common duckweed (*Lemna minor*) and hornwort (*Cerathophyllum demersum*). Water temperature did not change, although it must be stressed that were was a notably decreased oxygen concentration during the summer period. The lowest observed values oscillated around 1 mgO_2_l^−1^.

Before the construction of the retention basins, Smolnica, similar to Strumień Kiszewski before beaver reintroduction, was characterized by diverse morphometry and flow, the predominance of gravel in the bottom substrate, and very good physicochemical indexes (Table 1). Water crowfoot species (*Ranunculus* spp.), entered on the Critical List of Vascular Plants of Poland^57^, were also reported. In Smolnica, 50 years later, the natural channel profile was found only over a short, 3-km-long stretch; however, studies showed an almost twofold increase in width in relation to 1964 and a simultaneous shallowing of the watercourse. The most evident changes include a reduced share of gravel in the bottom substrate at an increase in the share of sand, the disappearance of water crowfoots (*Ranunculus* spp.), and the appearance of Canadian waterweed (*Elodea canadensis*). The temperature in the summer period also increased along with a simultaneous decrease in oxygen content.

### Changes in the macrozoobenthos species structure

**Figure 2:**
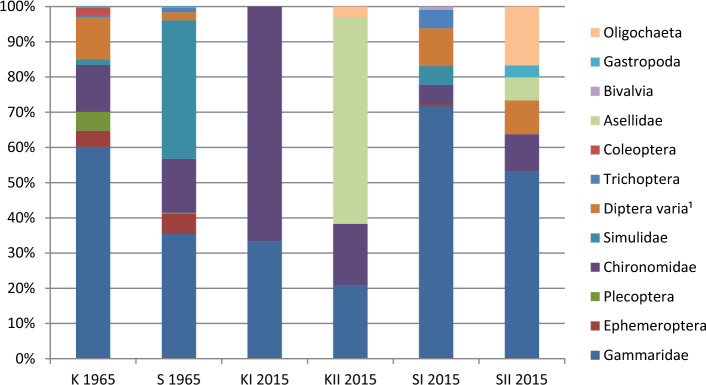
Changes in domination structure (D%) of macroinvertebrates in examined streams (^1^Diptera varia—Diptera, non-Chironomidae).

In 1964 in each of the streams, a total of 10 taxa were reported, found at large densities, considerably exceeding over 5000 ind.m^−2^ (Table 3). Among macrozoobenthos organisms, *Gammarus* species and black flies (Simuliidae) predominated, while other stenotopic groups were also present, such as stoneflies (Plecoptera), mayflies (Ephemeroptera), caddisflies (Trichoptera), in the absence of Asellidae and Oligochaeta (Fig. 2). Diversity indexes and cumulative BMWP scores in the investigated watercourses amounted to 0.6 and 51, respectively.

**Table 3:** Density (average ± SD) of macroinvertebrates, species diversity, and BMWP score at studied locations at two measurement dates.

No	Taxon	1964		2015			
		K	SM	KI	KII	SMI	SMII
		ind.m^−2^	ind.m^−2^	ind.m^−2^	ind.m^−2^	ind.m^−2^	ind.m^−2^
1	Gammaridae	3433 ± 1517	2463 ± 1667	23 ± 14	49 ± 13	1428 ± 228	571 ± 237
2	**Ephemeroptera**	257 ± 103	400 ± 273	0	0	12 ± 9	0
3	**Plecoptera**	306 ± 379	24 ± 48	0	0	0	0
4	Chironomidae	757 ± 1202	1060 ± 533	46 ± 7	41 ± 6	107 ± 69	113 ± 66
5	Simulidae	89 ± 113	2738 ± 3150	0	0	107 ± 119	0
6	Diptera varia	687 ± 495	158 ± 182	0	0	214 ± 174	103 ± 51
7	**Trichoptera**	33 ± 11	89 ± 95	0	0	104 ± 93	0
8	Coleoptera	123 ± 74	7 ± 13	0	0	0	0
9	*Asellus sp.*	0	0	0	138 ± 45	0	71 ± 61
10	Bivalvia	7*	7*	0	0	18 ± 15	0
11	Gastropoda	7*	7*	0	0	0	36 ± 28
12	Oligochaeta	0	0	0	7 ± 4	0	179 ± 59
Total		5685 ± 2272	6954 ± 4191	69 ± 21	235 ± 49	1990 ± 419	1073 ± 396
Number of taxa		10	10	2	4	7	6
Diversity H		0.57	0.59	0.28	0.46	0.44	0.60
BMWP		51	51	9	14	33	20

*In the paper of Iwaszkiewicz^48^, only single individuals of molluscs and snails are mentioned, therefore for these taxa, the smallest densities obtained during the own study are given. Reophilic, stenotypic taxa, with high environmental requirements are marked.

In 2015 in Strumień Kiszewski, only two taxa were identified within the area of the beaver dams (KI), found in 1/100 densities compared to the 1964 data (Table 3). This is reflected in the low values of the diversity index and BMWP values. Another situation was observed immediately below the dammed stretches (KII); four taxa were identified, including the waterlouse (*Asellus aquaticus*) and the sludge worm (*Tubifex tubifex*).

In Smolnica, depending on the distance from the dams, a total of 7 (SMI) and 6 taxa (SMII) were identified (Table 3). *Grammarus* predominated in both locations, although at much lesser densities than at the previous date, while at SMII oligochaetes were co-dominants.

**Figure 3:**
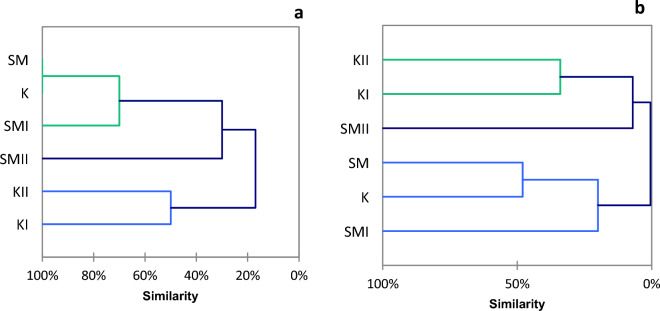
The faunistic similarity of macroinvertebrates in the qualitative (**a**) and quantitative (**b**) terms.

Cluster analysis showed a 100% qualitative faunistic similarity of the two unmodified habitats and an almost 50% quantitative similarity (Fig. 3). Another group identified in that analysis comprised modified stretches of Strumień Kiszewski (KI, KII), which differed markedly from the other stretches. Regarding the number of species, the similarity of these two habitats to the others amounted to 19%, while in terms of density, it was 6% in relation to SMII and 0% to the others. The SMI location showed a more remarkable similarity to the unmodified stretches.

### Changes in the ichthyofauna species structure

Analyses of ichthyofauna conducted in Strumień Kiszewski in 1964 and 1992 gave similar results. Three rheophilic species were identified, including the brown trout and the European brook lamprey, indicating low species diversity values. Based on the EFI+ index, the stream may be classified as the salmonid group in ecological class II. In 2015 no fish were found in the stretch of the watercourse within the area of beaver dams, while immediately below the dammed sections, four species were reported, with the dominance of stagnophilic. The only rheophilic species was the European brook lamprey.

**Figure 4:**
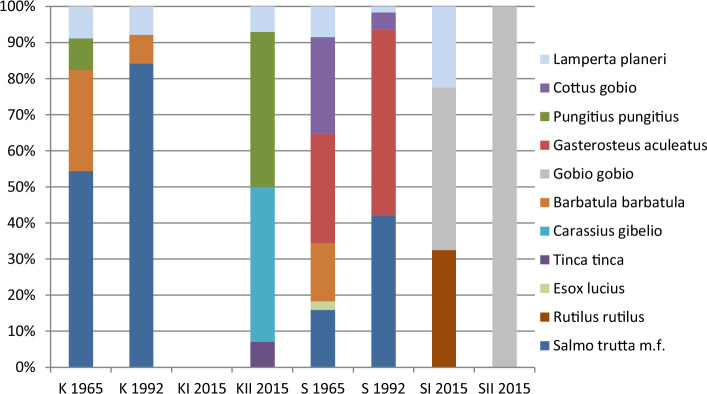
Changes in domination structure (D%) of ichthyofauna in examined streams.

During the first study on Smolnica, a total of seven species were reported, among which stenotypic fish of high environmental requirements predominated (Table 4, Fig. 4). In that period, the highest Shannon–Weaver index and EFI + values were also recorded. After more than 30 years, the population size of the brown trout increased, while that of the bullhead and stone loach decreased. In 2015 among indicator species, only the European brook lamprey was reported, while eurytopic fish appeared, i.e., the roach (*Rutilus rutilus*) and the gudgeon (*Gobio gobio*), which dominated the ichthyofauna (Fig. 4).

**Table 4:** Density (average ± SD or total) of ichthyofauna, species diversity, and European Fish Index at studied locations at three measurement dates.

No.	Species	Strumień Kiszewski				Smolnica			
		1964	1992	2015	2015	1964	1992	2015	2015
		K^1^	K^2^	KI^1^	KII^1^	SM^1^	SM^2^	SMI^1^	SMII^1^
		ind.*100 m^−2^				ind.*100 m^−2^			
1	**Brown trout** (***Salmo trutta m.fario*** **Linnaeus, 1758***)*	**9.4 ± 14.95**	**4**	No fish	**0**	**2.1 ± 1.18**	**4.2**	**0**	**0**
2	*Roach* *(Rutilus rutilus* Linnaeus, 1758*)*	0	0		0	0	0	1.7 ± 0.24	0
3	Pike *(Esox lucius* Linnaeus, 1758*)*	0	0		0	0.32 ± 0.16	0	0	0
4	Tench *(Tinca tinca* Linnaeus, 1758*)*	0	0		0.6 ± 0.21	0	0	0	0
5	Prussian carp *(Carassius gibelio* Bloch, 1784*)*	0	0		3.7 ± 0.71	0	0	0	0
6	Stone loach *(Barbatula barbatula* Linnaeus, 1758*)*	4.8 ± 3.37	0.4		0	2.1 ± 1.49	0	0	0
7	Gudgeon *(Gobio gobio* Linnaeus, 1758*)*	0	0		0	0	0	2.4 ± 1.07	1.4 ± 0.70
8	Three-spined stickleback *(Gasterosteus aculeatus* Linnaeus, 1758*)*	0	0		0	4.0 ± 2.45	5.1	0	0
9	Nine-spined stickleback (*Pungitius pungitius* Linnaeus, 1758)	1.5 ± 1.04	0		3.6 ± 0.99	0	0	0	0
10	**Bullhead** (***Cottus gobio*** **Linnaeus, 1758**)	**0**	**0**		**0**	**3.5 ± 0.88**	**0.5**	**0**	**0**
11	**European brook lamprey** (***Lamperta planeri*** **Bloch, 1784**)	**1.5 ± 0.49**	**0.4**		**0.6 ± 0.14**	**1.1 ± 0.64**	**0.2**	**1.2 ± 0.28**	**0**
	Total	17.2 ± 18.07	4.4		8.5 ± 1.56	13.1 ± 3.59	10.0	5.2 ± 1.49	1.4 ± 0.70
	Number of species	4	3		4	6	4	3	1
	Diversity	0.39	0.24	0	0.48	0.66	0.41	0.46	0
	EFI +	0.812 II	0.656 III	0.00 V	0.183 V	0918 I	0.571 III	0.441 IV	0.00 V

^1^Average ± SD density of catches; ^2^total density from the single catch. In 1992 only a single catch was made.

Reophilic, stenotypic species, with high environmental requirements are marked.

**Figure 5:**
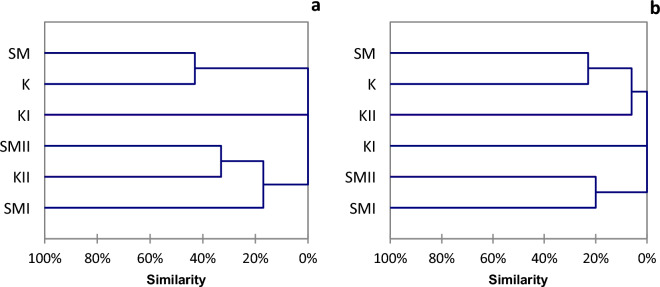
The faunistic similarity of ichthyofauna in the qualitative (**a**) and quantitative (**b**) terms.

In the case of ichthyofauna, cluster analysis showed a much lesser degree of faunistic similarity than in the case of macroinvertebrates (Fig. 5). Unmodified habitats (SM, K) were most similar qualitatively, as their similarity index was 43%, while they differed markedly from the other habitats. Another group comprised modified stretches KII and SMII. The section within the area of beaver dams differed from the other stretches by 100% due to the absence of fish.

## Discussion

Results of analyses of hydromorphological and physicochemical parameters clearly indicate a considerable habitat transformation, which occurred within a relatively short time after the reintroduction and uncontrolled increase in the beaver population in Strumień Kiszewski and after the construction of retention basins on Smolnica. At this point, it should be emphasized that both activities led to the fragmentation of watercourses and almost the same negative changes. In the studied streams and their catchment areas, no other negative impacts, such as sewage inflow, agriculture, or forest management, could change the environmental conditions—there are no other pressures that affect the tested parameters.

Most cited authors are of the opinion that beavers in flowing waters cause far-reaching changes in hydromorphological, geomorphological, and physicochemical conditions; however, a definite assessment of these changes—whether they are positive or negative—raises certain controversies^33,34,36,58–63^. According to the authors of this study, Strumień Kiszewski is an example of lotic habitat degradation. The activity of a large beaver population destroyed the transitional zone, reduced the heterogeneity of microhabitats, and caused silting of gravel pits and oxygen depletion in the summer.

The effect of watercourse partitioning and damming is relatively well-known, and the opinions of hydrobiologists on the subject are unanimous. Despite numerous studies and increased environmental awareness, small watercourses are still perceived as those which may be easily transformed for economic purposes^4^. As a result, streams are eliminated from the landscape due to the construction of ponds and retention basins or drainage and elimination of washlands. An example of such actions may be provided by Smolnica, which over a greater part of its course has been transformed into fishing ponds and retention basins, while at short stretches of flowing waters, environmental conditions are strongly modified about the period before damming. Retention reservoirs constructed on Smolnica have caused watercourse fragmentation with all its negative consequences.

Deterioration of environmental conditions in the watercourses analyzed was manifested in substantial modifications affecting macroinvertebrates and ichthyofauna.

In Strumień Kiszewski, the decrease in the number of invertebrate species was as high as fivefold, along with almost complete elimination of rheophilic stenobionts and a simultaneous appearance of previously unreported eurytopic organisms. A similar direction of changes caused by many beaver dams on a small watercourse was presented in a study by Rowe^34^, who observed a decrease in species diversity of aquatic macroinvertebrates, an increase in diversity of terrestrial organisms, and changes in trophic relations. In turn, Strzelec et al.^63^, Bylak et al.^64^, and Bylak and Kukuła^39^ reported a positive effect of beavers on invertebrate species diversity when investigating upland and mountain streams, thanks to the alternating sections of flowing and stagnant waters. The contrary results and conclusions regarding the influence of beaver dams most likely result from different landscape features, among which the slope and the associated flow velocity are decisive.

In Smolnica, similar changes in the species structure of benthic macroinvertebrates were also recorded; however, they were not as drastic as in Strumień Kiszewski. The extensive literature on the subject shows that dams, as well as all hydraulic structures, have a negative effect on the number of species, density, and biomass of benthic invertebrates^26,32,65–67^. The fast water current immediately below the damming washes out aquatic organisms, while sanding and silting of the bottom on further reach results in transforming their species composition. In the summer, an increase in temperature and a decrease in oxygen content limit the potential for the survival of particularly sensitive organisms. Townsed et al.^68^, Genkai-Kato et al.^69^, and Liu et al.^67^ found a definite negative correlation between temperature and oxygen in the summer and the density of Plecoptera and Ephemeroptera. Among invertebrate fauna, stoneflies (Plecoptera) are especially valuable in terms of high environmental requirements^70^. Kazanci^71^ also showed a lack of tolerance of species from the genus *Simulium* to an increased temperature and the absence of vegetation in the transitional zone.

The results presented in this study are consistent with the observations mentioned above. Immediately below the damming on Smolnica, no representatives of Plecoptera, Ephemeroptera, or Simuliidae were found, while previously unreported taxa, i.e., Asellidae, Sphaeriidae, and Oligochaeta, were recorded.

The species composition of ichthyofauna in the watercourses analyzed in the 1960s may be considered as reference for clean lowland streams with the dominance of species with high environmental requirements^48^. The most endangered fish species of those described in this paper area are bullhead (*Cottus gobio*), European brook lamprey (*Lamperta planeri*), and native brown trout (*Salmo trutta m. fario*)^6^.

The study, repeated after 50 years under disturbed equilibrium, showed a complete transformation of assemblages benefitting eurytopic species. No fish were found in Strumień Kiszewski within the area of beaver ponds, while below the damming, out of all the indicator species, only the European brook lamprey was caught. Simultaneously stagnophilic species appeared, which is consistent with observations reported by Hägglund and Sjöberg^72^, who confirmed that adequate environmental conditions for cyprinids are created after the construction of a beaver dam.

The complete disappearance of the brown trout, reported during this study, clearly indicates a negative role of beavers; however, analysis of the literature on the subject does not provide grounds for such definite conclusions^35^. Numerous authors think that the presence of beaver dams has a positive effect on ichthyofauna, both qualitatively and quantitatively. Kemp et al.^33^ reported that this is the opinion of most of the 49 experts from North America and Europe who were surveyed. Thanks to their activity, beavers indirectly cause an increase both in watercourse fertility and in the number of ecological niches and shelters, increasing the fish population^35,37,43,60,62,73,74^. Even more, far-reaching conclusions were drawn by Grasse^58^ and Andonaegui^59^, who stated that beaver activity has a positive effect on the growth and production of salmonids while reintroducing that species may be a cost-effective method to improve habitats for salmon and lake brown trout. In upland and mountain streams of south-eastern Poland, the greatest density, percentage, and body size of brown trout were observed within beaver ponds, while in fast-flowing stream sections, only fry was found^60,74^. The same study shows that after the introduction of beavers, the condition of ichthyofauna in a stream degraded by forest tending operations improved considerably.

Virbickas et al.^61^, when investigating Lithuanian lowland streams, found no differences in terms of the number of fish species or the diversity indicator between stretches located upstream and downstream of beaver dams; however, eurytopic species such as the roach and the perch, in contrast to litophilic species, dominated primarily upstream of the dam.

Negative opinions on beaver activity refer mainly to water stagnation, deterioration of physicochemical parameters, disturbed migration, and reduced areas of spawning grounds due to their sanding^33^. In lowland streams, a negative effect of beaver dams was observed in the results of sea trout stocking. After watercourse damming, fewer fry were caught compared to the period with no beaver activity^42^. Beaver dams also prevent upstream fry migration while causing deterioration of environmental conditions. Hägglund and Sjöberg^72^ thought that beaver recolonization of small watercourses leads to the depletion of natural spawning grounds for salmonids. Pollock et al.^40^ reported a reduction of brown trout fry amounting to as much as 60%. No redds are found over the entire length of backwater upstream of the dams^75^.

Results from the cited literature sources show that the degree of the negative impact of beavers on ichthyocenoses depends first of all on the location and character of the watercourse as well as species density and the number of dams over a given stretch of the river, which has to be taken into consideration in species protection plans. Research results confirm that this impact could definitely be negative in the case of small lowland watercourses at a sequence of several beaver damming.

In Smolnica, from the stenotypic species, only the European brook lamprey was found at least 1 km downstream of the damming. Only gudgeons and roaches were found over the entire stream stretch. Depletion of lithophilic species is observed primarily in the case of river stretches upstream of damming^5,26^. The roach, being eurytopic, is frequently dominant in degraded watercourses, while the degree of dominance may be an indicator of the level of aquatic system transformation^1,6,27,29,76^. The brown trout and the bullhead are very sensitive to changes in environmental parameters^77–81^, which was confirmed by the results of studies conducted by the authors of this paper. Here an increase in water temperature, sanding of gravel pits, as well as a reduced mosaic pattern of microhabitats following the construction of retention basins has resulted in the disappearance of these two fish species.

The results presented in this paper allow us to confirm the formulated hypotheses. Both the presence of retention reservoirs and the uncontrolled growth of the beaver population, in the case of small lowland streams cause far-reaching, negative changes in abiotic and biotic factors.

In view of the authors’ own research results, presented against the backdrop of world literature, there is a question of the validity of unlimited one-species protection and the construction of as many, large in relation to the size of the watercourse, retention reservoirs on such valuable natural objects. Any decision on the execution or rejection of a planned action interfering with aquatic ecosystems needs to be based on assessing benefits and losses connected with the attained objectives, including nature conservation. When a decision is made, it is necessary to select solutions, making it possible to reach assumed goals while simultaneously protecting all nature resources^3,82^. In the authors’ opinion, adopting a holistic approach to the problem, comprising all its aspects, would have prevented the damage. In the case of Strumień Kiszewski, monitoring the beaver population may have facilitated actions limiting its growth and, as a result, would have provided grounds for a significant reduction of the resulting habitat transformation. Similarly, constructing only one retention basin on Smolnica would have combined priorities of technical small retention and protection of valuable hydrobiont species.

We hope that the case described in this study will introduce changes in the public outlook on nature conservation and will prevent similar erroneous actions in the future.

## Data Availability

The datasets generated and analyzed during the current study are available from the corresponding author on reasonable request.
